# Stromagenesis and cancer‐associated fibroblast heterogeneity in primary tumors and metastasis: focus in non‐small cell lung cancer

**DOI:** 10.1002/2211-5463.70102

**Published:** 2025-09-04

**Authors:** Alejandro Bernardo, Natalia Díaz‐Valdivia, Patricia Fernández‐Nogueira, Jordi Alcaraz

**Affiliations:** ^1^ Unit of Biophysics and Bioengineering, Department of Biomedicine University of Barcelona Spain; ^2^ Institute for Bioengineering of Catalonia (IBEC) The Barcelona Institute for Science and Technology (BIST) Spain; ^3^ Thoracic Oncology Unit Hospital Clinic Barcelona Spain; ^4^ Centro de Investigación Biomédica en Red de Enfermedades Respiratorias (CIBERES) Instituto de Salud Carlos III Madrid Spain

**Keywords:** cancer‐associated fibroblasts, desmoplasia, metastasis, myofibroblasts, non‐small cell lung cancer, stromagenesis

## Abstract

Non‐small cell lung cancer (NSCLC) is the most common lung cancer type and one of the deadliest neoplasias worldwide. NSCLC is histologically classified into adenocarcinoma, squamous cell carcinoma, and other less frequent subtypes. Both subtypes and other solid tumors are increasingly regarded as abnormal organs, highlighting the critical role of the desmoplastic tumor stroma rich in cancer‐associated fibroblasts (CAFs) in driving tumor progression and therapeutic resistance. This tumor stroma resembles a chronic fibrotic wound and is largely formed by activated/myofibroblast‐like α‐SMA+ CAFs (myCAFs), which are strongly associated with immunosuppression and poor prognosis. Despite the dominance of the myCAF phenotype, we reported a decade ago phenotypic alterations in NSCLC with a strong dependence on the histologic subtype. Subsequent studies using functional assays, single‐cell techniques, and *in vivo* models have refined these initial observations, enhancing our understanding of the biology of both normal fibroblasts/myofibroblasts and CAFs in NSCLC and other cancer types, including their origins, subclassification, and physiopathologic functions. Notably, increasing evidence supports that CAFs can exhibit tumor‐restraining or tumor‐promoting effects, and current therapeutic efforts aim to shift the balance towards tumor‐restraining phenotypes. Here, we review major advances in our understanding of tumor stromagenesis and CAF heterogeneity in both primary tumors and metastasis, including emerging consensus, with a special focus on NSCLC and its frequent dissemination to the brain. We also highlight the critical role of smoking through epigenetic reprogramming of the TGF‐β/SMAD3 pathway. These advances are beginning to delineate how CAF heterogeneity depends on the stage and histologic subtype in NSCLC.

## Graphical abstract

Cancer‐associated fibroblasts (CAFs) play different roles in NSCLC progression and metastasis. Their heterogeneous origins and phenotypes shape the tumor microenvironment and influence metastatic spread. This review highlights NSCLC CAF subtypes, their potential cellular sources, and their differential distribution across primary tumors and distant organs, emphasizing their context‐specific functions in stromal remodeling and immune modulation.

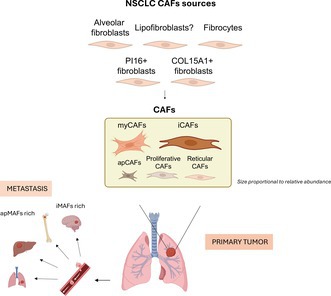

**Abbreviations**
apCAFs: antigen‐presenting cancer‐associated fibroblasts
a‐SMA: alpha‐smooth muscle actin
CAFs: cancer‐associated fibroblasts
CTCs: circulating tumor cells
DAMP: damage‐associated molecular patterns
ECM: extracellular matrix
EMT: epithelial‐to‐mesenchymal transition
iCAFs: inflammatory cancer‐associated fibroblasts
iMAFs: inflammatory metastasis‐associated fibroblasts
LUAD: lung adenocarcinoma
LUSC: lung squamous cell carcinoma
MAFs: metastasis‐associated fibroblasts
mIHC: multiplex immunohistochemistry
MSCs: mesenchymal stem cells
myCAFs: myofibroblast‐like cancer‐associated fibroblasts
myMAFs: myofibroblast‐like metastasis‐associated fibroblasts
NSCLC: non‐small cell lung cancer
scRNAseq: single‐cell RNA sequencing
TAFs: tumor‐associated fibroblasts
TIMP1: tissue inhibitor of metalloproteinase‐1
TNM: Tumor‐Node‐Metastasis

Lung cancer is the deadliest cancer type in both women and men worldwide, with an average 5‐year survival of 25% [1], thereby posing a major biomedical challenge. Although smoking is the primary risk factor, lung cancer can also develop in nonsmokers without any identifiable predisposing condition [2]. Histologically, most patients are diagnosed as non‐small cell lung cancer (NSCLC), which is further classified into adenocarcinoma (LUAD), squamous cell carcinoma (LUSC), and other less frequent subtypes [3]. LUSC is strongly associated with smoking and frequently arises within the proximal airways, whereas LUAD is the most common subtype in non‐smokers and is often detected in distal pulmonary sites [4]. Despite recent advances in early detection and therapeutic options, the prognosis of NSCLC remains unfavorable due to its late diagnosis and high metastatic dissemination to secondary organs such as the brain, bone, lung, and liver [5, 6]. Notably, although all these NSCLC subtypes are epithelial in origin (i.e., carcinomas), it is now clear that the tumor stroma surrounding cancer cells plays a critical role in driving tumor progression and promoting therapeutic resistance [7, 8, 9]. Accordingly, understanding how this tumor‐supportive stroma is formed (process called stromagenesis), how it contributes to this fatal disease, and how it can be targeted therapeutically is receiving increased attention.

Pathologists have long recognized that the stroma of most solid tumors is highly desmoplastic, owing to its striking similarities with fibrotic wounds [10], supporting the interpretation of tumors as “wounds that do not heal” [11]. This desmoplastic stroma is formed by fibroblasts, vascular cells, immune cells, nerve fibers, and a collagenous‐rich interstitial extracellular matrix (ECM) [7, 12], although fibroblasts are the most abundant stromal cell type [7, 13]. Cancer‐associated fibroblasts (CAFs), also known as tumor‐associated fibroblasts (TAFs), were first described as a population rich in activated/myofibroblast‐like fibroblasts, owing to its marked upregulation of the contractility marker α‐SMA, which had also acquired strong tumor‐promoting functions [14, 15]. In NSCLC, even though most CAFs are α‐SMA+, we reported a decade ago that they exhibit additional phenotypic alterations that depend strongly on the histological subtype [16]. Subsequent studies have unraveled the functional complexity of CAFs, including evidence that they can be tumor‐restraining or tumor‐promoting [17, 18]. Similarly, recent advances in single‐cell techniques, including scRNAseq and multiplex immunohistochemistry (mIHC), have shed light on the biology of normal fibroblasts/myofibroblasts and on the heterogeneity and potential sources of CAFs [19, 20].

Although a complete consensus on the origins, subtypes, and functions of CAFs in NSCLC is still lacking, some biological features of CAFs are shared across different cancer types, whereas others are lung cancer‐specific and may even depend on the histologic subtype [21, 22]. Furthermore, disseminated cancer cells require support from a stroma rich in fibroblasts in metastatic sites; yet, these metastasis‐associated fibroblasts (MAFs) are much less characterized than CAFs [23]. This review summarizes recent advances in our understanding of tumor stromagenesis and CAF heterogeneity in the context of primary tumors and metastatic settings, including emerging consensus, with a special focus on NSCLC and its common dissemination to the brain. We also highlight major challenges and open questions in the field. Other aspects of the complex biology of CAFs, including their contributions to immunomodulation, metabolism, drug resistance, and therapeutic opportunities, are covered elsewhere [24, 25, 26, 27, 28].

## Key insights into fibroblast and myofibroblast biology

There is substantial information on the biology of normal fibroblasts and myofibroblasts that may help understand key aspects of CAFs, including their origins and potential tumor‐restraining and tumor‐promoting functions. Here, we summarize some of these critical advances. Surprisingly, despite its first description by Rudolf Virchow nearly two centuries ago [29], no specific fibroblast markers have been reported. Instead, fibroblasts are defined by a combination of spindle‐shaped nuclear and cell body morphologies, location within connective tissue, lack of lineage markers for other major cell types (epithelial, endothelial, and immune cells), and positivity for different markers, although none of them is fibroblast‐specific [30, 31]. A consensus on the identity and number of such nonspecific markers is lacking, but widely used protein markers include vimentin, type I collagen, and PDGFR‐α, whereas α‐SMA remains the gold standard for activated fibroblasts/myofibroblasts [22, 32, 33]. HIC1 is an emerging marker for myofibroblast progenitors [32], but its validation in CAFs is awaiting.

Regarding their functions, fibroblasts were initially identified based on their ability to produce new collagen‐rich connective tissue upon injury through controlled ECM remodeling and transient acquisition of an α‐SMA‐dependent contractile myofibroblast phenotype [17]. It became subsequently clear that fibroblasts can perform additional tasks, including providing positional/niche cues for tissue‐resident stem cells, and acting as progenitors of either more ECM‐producing fibroblasts or a variety of specialized tissue‐specific mesenchymal cells [34]. Fibroblasts' progenitor potential is highlighted by their efficiency in generating induced pluripotent stem cells, supporting their common identification with tissue‐resident mesenchymal stem cells (MSCs) [17]. Fibroblasts are also increasingly pointed out as critical regulators of immunity [34]. Another emerging fibroblast function is the sensitivity to danger‐associated molecular pattern (DAMP) signals, which are intracellular macromolecules released from damaged cells [34, 35]. Such DAMP sensitivity may be particularly relevant in solid tumors exhibiting extensive necrosis [36]. All these multifaceted roles make fibroblasts essential for tissue support, repair, and homeostasis [7, 37].

Most known fibroblast functions become apparent only upon their activation. Yet, fibroblasts remain in a quiescent/resting state in normal physiologic conditions, in which their specific functions are much less defined, although it is likely that they involve fibroblast's “readiness” to effectively restore tissue homeostasis upon injury [17]. Fibroblast activation can be mediated by several soluble cues, including TGF‐β1, WNT, PDGF, and CTGF, which ultimately increase the expression of α‐SMA; however, TGF‐β1 is considered the most potent α‐SMA inducer [32, 38, 39]. Most tissue TGF‐β1 is secreted in an inactive form that binds to the collagenous ECM, thereby remaining ready to facilitate repair [36, 40], in line with the expected “readiness” of resting fibroblasts. Additional fibroblast activation factors are emerging in physiopathologic conditions, such as the extracellular glycoprotein SPP1/osteopontin [21, 39, 41]. Alternatively, fibroblasts can escape their quiescent/resting state through inflammatory modulators such as IL‐1, IL‐6, and TNF‐α [30, 34]. However, it remains ill‐defined how these profibrotic and pro‐inflammatory cues modulate fibroblast behavior when acting in combination, as expected in more complex microenvironments as in tumors.

Normal myofibroblasts were first described in healing rat wound granulation tissue as cells that share morphological features of normal tissue‐resident fibroblasts and contractile smooth muscle cells [42]. They play a crucial role in tissue repair by driving the transient deposition of a collagen‐rich scar. Myofibroblasts are also key in eliciting a persistent contracting scar tissue in organ fibrosis [32]. Therefore, enhanced α‐SMA‐dependent contraction, collagen deposition, and associated supermature adhesions are functional hallmarks of the fully differentiated myofibroblast phenotype [32]. However, the acquisition of this phenotype is considered the final state of a gradual maturation process, which may account for the variety of myofibroblast‐like subtypes reported in scRNAseq studies in physiopathologic conditions, including solid tumors.

Regarding their origin, most tissue‐specific fibroblasts emerge from the mesoderm during embryonic development to populate numerous organs, being the heart, skin, skeletal muscle, lungs, and liver the most fibroblast‐rich [34]. These tissue fibroblasts display distinct positional and organ‐specific transcriptional signatures [43]. Yet, recent cross‐tissue scRNA‐seq analyses in mouse and human organs have identified two universal fibroblast subtypes in physiologic conditions: PI16+ (adventitial) and COL15A1+ (parenchymal), which have the capacity to produce collagenous interstitial ECM and basement membrane components, respectively [21, 44]. scRNAseq analyses have also reported a ubiquitous population of LRRC15+ myofibroblasts upon activation in different organs, including the lungs [44, 45].

In the context of pulmonary tissue, adventitial fibroblasts reminiscent of the universal PI16+ subtype have been reported near conducting airways and blood vessels, and have also been referred to as matrix fibroblasts [31]. Additional pulmonary fibroblast subtypes have been described based on their specific location and/or function, including alveolar fibroblasts (located within distal parenchymal regions) and pneumocyte‐supporting lipofibroblasts [31, 34]. Pulmonary myofibroblasts have also been extensively reported upon injury and in profibrotic conditions [46, 47, 48]. These myofibroblasts are generally positive for the classical contractility marker α‐SMA and the emerging fibrotic marker CTHRC1 [47, 49], and resemble the LRRC15+ population. A list of developing consensus markers of normal pulmonary fibroblast subtypes is shown in Table 1 [22, 34]. Although all these fibroblast subtypes have different anatomical locations and unique expression profiles, some of them can interconvert, illustrating fibroblast plasticity [31].

**Table 1:** Key transcriptional markers for normal/progenitor pulmonary fibroblast subtypes.

	Gene	Ref cross‐tissue	Ref lung
Adventitial fibroblasts (PI16)	*PI16*	[21], [34]^a^	[22], [31]^a^
	*CCL11*	[34]^a^	
	*COL1A1*	[34]^a^	
	*PDGFRA*	[34]^a^	
	*CD34*	[21]	[22]
	*LEPR*		[22]
	*PLA2G2A*		[22]
	*SFRP2*		[31]^a^
	*SERPINF1*		[31]^a^
	*MFAP5*	[21]	[31]^a^
Parenchymal fibroblasts (COL15A1)	*COL15A1*	[21], [34]^a^	
	*FGF7*	[21]	
	*APOD*	[21]	
Alveolar fibroblasts	*ADH1B*	[21]	[22]
	*LIMCH1*	[21]	
	*A2M*	[21]	
	*NPNT*		[34]^a^, [31]^a^
	*ITGA8*		[34]^a^, [31]^a^
	*MME (CD10)*		[22]
	*FIGF (VEGFD)*		[22]
	*FGFR4*		[22]
	*ELN*		[22]
Lipofibroblasts	*PLIN2*		[34]^a^
	*COL4A1*		[34]^a^
	*APOE*		[31]^a^
	*FST*		[31]^a^

^a^ In fibrotic conditions (not cancer).

## Mechanisms of stromagenesis in the primary tumor

The formation of the tumor stroma rich in CAFs (stromagenesis) is far from being completely defined, yet there is evidence that tumor nests and their surrounding stroma co‐evolve through complex crosstalks involving different cell types. Likewise, stromagenesis is strongly influenced by the epigenetic reprogramming of CAFs [17, 24, 50]. Here, we summarize recent findings on the key processes underlying stromagenesis, including those that are specific for NSCLC.

### Tumor stromagenesis as tissue repair gone awry

An initial accumulation of cancer cells may elicit a stromal host response similar to tissue repair settings [7, 13]. In most cases, the immune system effectively eliminates transformed cells as part of the host defense response [26]. In other occasions, the host response elicits a reactive stroma rich in activated fibroblasts that encapsulates these early precancerous lesions, effectively restraining tumor progression [17, 30]. Consistently, a parallel organization of collagen fibers was associated with better prognosis in LUAD. Likewise, stromal encapsulation is associated with better outcome in liver metastasis [51]. The protective function of the stroma may last our lifetime, as revealed by careful postmortem cross‐tissue examination showing several *in situ* carcinomas or precursor lesions in different organs that never developed into cancer [7]. However, some of these early cancerous lesions may escape the protective stroma through processes that remain poorly understood [26]. Nonetheless, different contexts can facilitate the stromal switch from tumor‐restraining to tumor‐permissive or even tumor‐promoting, including the existence of a previous injury/wound, aging, chronic inflammation, and/or epigenetic reprogramming through heterotypic cancer cell‐stromal cell interactions [7, 26, 52, 53]. Among these tumor‐permissive processes, sustained inflammation appears to be particularly relevant in CAFs, since cross‐tissue scRNAseq analysis of fibroblasts from different physiopathologic conditions, including tumors, revealed an early increase in the proportion of myofibroblasts in inflammation prior to malignancy [21]. In the pulmonary context, smoking is another major driver of pathologic inflammation and stromal epigenetic reprogramming [24, 54, 55]. Thus, normal pulmonary fibroblasts exposed to cigarette smoke particles exhibit increased contractility, enhanced ECM production, and altered cytokine secretion profiles [56], eliciting a fibroinflammatory environment that resembles early fibrosis and may facilitate the development of preneoplastic lesions [57]. All these processes ultimately facilitate the aberrant persistence of a tumor stroma rich in activated CAFs.

**Fig. 1:**
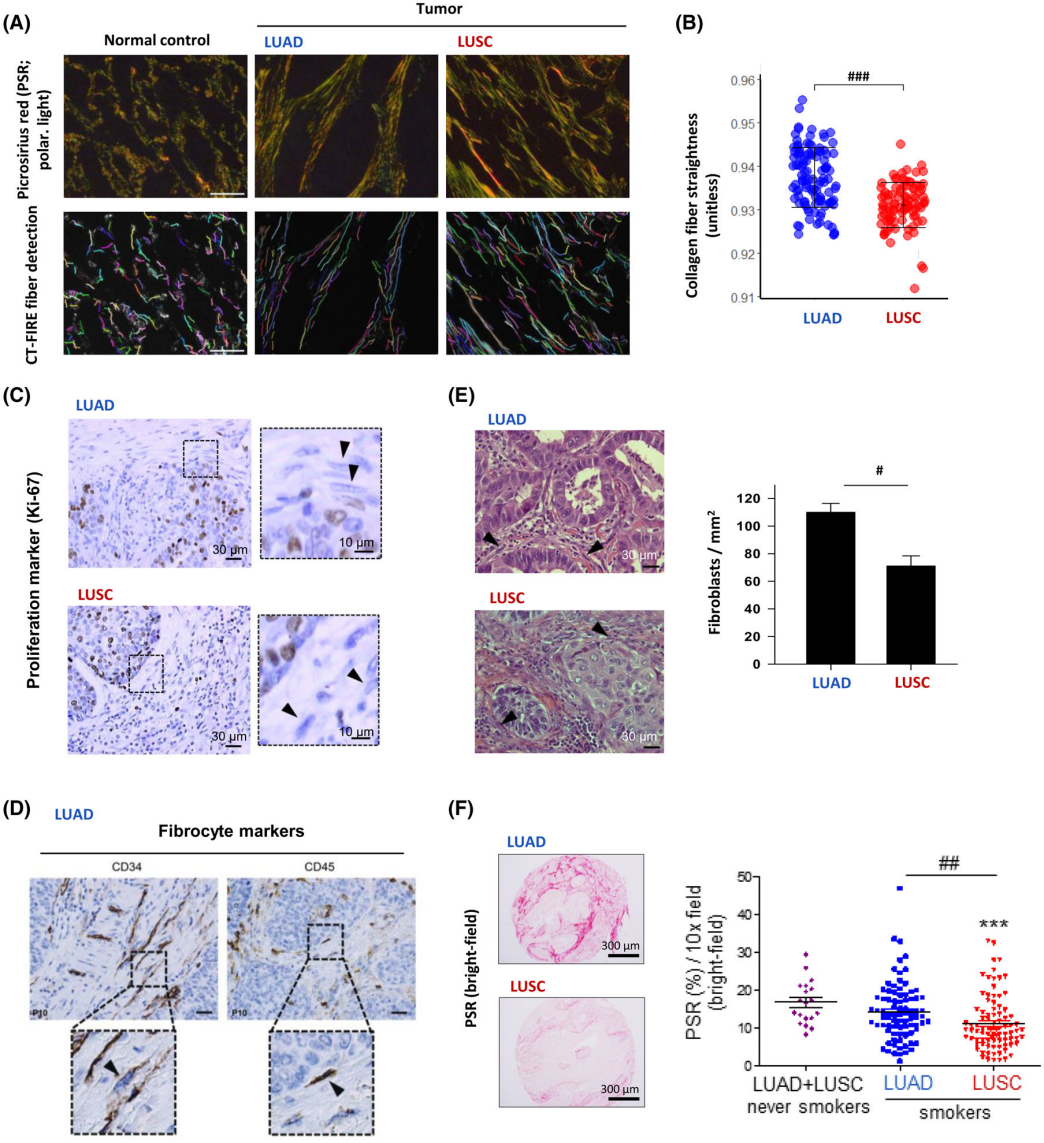
Histological hallmarks of the desmoplastic stroma in LUAD and LUSC. (A) Representative images of picrosirius red stains of fibrillar collagens imaged with polarized light (top) and corresponding single collagen fiber reconstructions by CT‐Fire software (bottom) of control pulmonary tissue, LUAD, and LUSC. Single collagen fibers are labeled with different colors. Reprinted from [95] with permission. Scale bar, 100 μm. (B) CT‐Fire quantification of collagen fibers straightness in LUAD and LUSC samples. Each dot represents the average of each patient. Reprinted from [95] with permission. ###, *P* < 0.001. (C) Representative histological stains of the proliferation marker ki‐67 in LUAD and LUSC tissue sections and zoom‐in selections. Black arrows point out individual CAFs. Reprinted from [56] with permission. (D) Representative histological stains of fibrocyte markers CD34 (left) and CD45 (right) in LUAD tissue sections and zoom‐in selections. Black arrows point out positive non‐endothelial mesenchymal cells. Reprinted from [60] with permission. (E) Quantification of CAF number density in LUAD and LUSC tissue sections. Black arrows point to CAFs identified by morphometric criteria in representative H&E images. Reprinted from [16] with permission. #, *P* < 0.05. (F) Representative histological images from tissue microarrays of picrosirius red stains of LUAD and LUSC tissue sections and quantification of the corresponding percentage of positive area, referred to as PSR%. LUAD and LUSC samples were further subclassified according to smoking status. Reprinted from [54] with permission. ***, *P* < 0.005 with respect to never‐smokers; ##, *P* < 0.01.

Of note, tumors thrive in the context of such desmoplastic stroma, which is roughly characterized by a pathologic accumulation of local and/or recruited fibroblasts in the background of an excessive deposition of fibrillar collagens (Fig. 1A,B), fibronectin, and other fibrotic ECM components. It is commonly assumed that such large fibroblast accumulation simply relies on their proliferation [13, 17], as in normal tissue repair [34]. However, molecular analyses of patient samples have shown that only a very small fraction of CAFs are positive for proliferation markers in NSCLC and other solid tumors [58, 59, 60] (Fig. 1C). A possible explanation could be that fibroblast proliferation occurs at very early stages as part of an inflammatory host response [21, 32, 61], although direct experimental evidence is lacking. In addition to proliferation, recruitment of both local fibroblasts through enhanced migration or circulating bone marrow‐derived progenitors (fibrocytes) is considered another contributor to CAF accumulation [17, 30]. Consistently, there is direct evidence of fibrocytes in NSCLC [62] (Fig. 1D). Some CAFs may also arise through the transdifferentiation of local tissue cells, including endothelial cells and perivascular cells (i.e., pericytes), although these contributions are expected to be minor [17, 30]. New preclinical models that enable long‐term monitoring of the dynamic formation of the tumor stroma are needed to dissect the detailed processes underlying CAF expansion and their sustained activation.

CAF accumulation is thought to be largely driven by growth factors released by cancer cells and/or infiltrating immune cells, such as members of the TGF‐β, PDGF, FGF, and VEGF families [17, 60, 63]. Among them, TGF‐β1 appears to be particularly relevant in CAFs because, in addition to being a potent inductor of fibroblast activation in physiopathologic conditions [32, 38], it is commonly overexpressed in solid tumors [64]. However, TGF‐β1 is largely cytostatic and hinders fibroblast migration [65, 66], supporting that it may be more relevant at later tumor stages. In contrast, PDGF is a strong stimulator of CAF migration [67]. Indeed, it is likely that the concentration of these soluble factors evolves during tumorigenesis as in tissue repair, and that TGF‐β1 abundance becomes ultimately dominant. In addition to soluble factors, biomechanical cues arising from the collagen‐rich stromal ECM [61] may enhance CAF activation and subsequent fibrosis [30, 46], in cooperation with TGF‐β1 [40, 68]. The relevant role of mechanical cues is further illustrated by the fact that TGF‐β1 activation from its latent protein complex is mediated by extracellular ECM mechanical tension and/or intracellular contractility [32].

### Histology‐dependent mechanisms of stromagenesis in NSCLC

Both CAF number density and expression of myofibroblast markers are higher in LUAD than LUSC [16, 54] (Fig. 1E), supporting that stromagenesis processes are histology‐dependent. Consistently, *in vitro* studies using hydrogels with tunable elasticity revealed that matrix stiffening alone is sufficient to enhance fibroblast accumulation through increased proliferation and decreased apoptosis, and that this mechanobiology process was critical for the expansion of CAFs in LUSC patients; in contrast, the expansion of LUAD‐CAFs required mitogenic cues from soluble factors [16]. Moreover, lung CAFs are epigenetically reprogrammed through global DNA demethylation and selective DNA hypermethylation of important genes, including the profibrotic transcription factor SMAD3, which is a critical effector of the canonical TGF‐β1 pathway [62]. Subsequent work demonstrated that the epigenetic repression of SMAD3 through DNA promoter hypermethylation was higher and functionally relevant in LUSC compared to LUAD, owing to the higher exposure to cigarette smoking particles in LUSC. Consistently, collagen deposition in patient samples was higher in never‐smokers than in smokers, with LUSC exhibiting the lowest levels [54] (Fig. 1F). In addition, migration studies in 3D type I collagen hydrogels revealed that fibroblasts exhibiting high SMAD3, as in LUAD‐CAFs, exhibited a migratory advantage in the absence of exogenous TGF‐β1 through a glucose‐dependent mechanism [65]. This suggests that early recruitment via increased fibroblast migration may significantly contribute to the higher CAF density in LUAD. All these findings have unraveled how smoking‐induced epigenetic reprogramming of CAFs controls stromagenesis in NSCLC in a histotype‐dependent manner.

## Cancer‐associated fibroblasts (CAFs)

### CAF definition and common origins

A previous consensus statement described CAFs similarly to normal fibroblasts, yet in the context of a tumor, with the addition of lacking the mutations found in cancer cells. The latter addition is relevant because it excludes cells undergoing a marked epithelial‐to‐mesenchymal transition (EMT) [30, 52]. However, CAFs exhibit important differences from normal fibroblasts and myofibroblasts. First, fibroblast activation is transient in physiological conditions, whereas it remains chronic and strongly associated with tumor progression in solid tumors [7, 13]. The mechanisms underlying this chronic activation are poorly understood, yet it is conceivable that they include the epigenetic reprogramming of CAFs, in which TGF‐β1 and ECM stiffening play major roles [24, 32, 69]. Second, CAFs can exhibit contrasting effects on tumor progression depending on their phenotype, in which CAFs exhibiting a myofibroblast‐like phenotype are generally considered tumor‐promoting, whereas those exhibiting a normal‐like phenotype are regarded as tumor‐restraining [17, 18, 70].

Although our understanding of tumor protective CAFs is very limited [18], the protective role of normal‐like fibroblasts has been associated with their ability to preserve tissue architecture [7] and to mediate effective immune responses [26]. ISLR/Meflin is a marker of undifferentiated MSCs [71] that has been pointed out as a potential mediator of tumor‐restraining CAFs in pancreatic ductal adenocarcinoma and fibrosis [72], although the underlying mechanisms and potential functions in NSCLC and other cancer types remain undefined. Conversely, the aberrant and chronic activation of CAFs elicits an excessive production of collagenous ECM (Fig. 1A) and matrix crosslinking enzymes that profoundly alter normal tissue organization and promote T‐cell exclusion [22, 61, 73]. In addition to these insoluble ECM cues, CAFs produce a variety of soluble factors that may enhance the maintenance of cancer stem cells and promote immunosuppression, angiogenesis as well as cancer cell invasion, growth, metastasis, and therapeutic resistance. These effects are mediated through a complex secretome, including TGF‐β1, IL‐6, HGF, and TIMP‐1, which elicit an aberrant activation of oncogenic pathways such as STAT3, Wnt/β‐catenin, and Notch [17, 28, 30, 74]. Consistently, high levels of standard markers of either CAF activation or CAF number density in tumor biopsies have been associated with bad prognosis in NSCLC and other solid tumors independently of the Tumor‐Node‐Metastasis (TNM) staging system, whereas tumors enriched in normal‐like fibroblasts exhibit longer survival [13, 21, 22, 75, 76].

### CAF heterogeneity: emerging consensus

In line with the analogy of tumors as wounds that do not heal, CAFs exhibiting a myofibroblast‐like phenotype (commonly referred to as myCAFs) are dominant in solid tumors [21, 60]. Moreover, previous work in pancreatic cancer identified an abundant population of CAFs rich in IL6 and other inflammatory markers [59] and a minor subset of CAFs expressing antigen‐presenting genes [77], referred to as iCAFs and apCAFs, respectively. myCAFs were observed in close proximity to cancer cells, whereas iCAFs were predominantly found in more distal locations [59], although the universality of this distribution is unclear. More recent single‐cell molecular analyses reported transcriptional signatures characteristic of lymphoid structures in a very minor CAF population, which was labeled as reticular CAFs [22, 60]. Likewise, markers of proliferation were found in a narrow subset of CAFs in different cancer types [21, 60]. All these CAF subtypes have been reported in NSCLC and most other solid tumors [21, 22, 58, 60], and there is emerging consensus regarding some of their transcriptional markers (Table 2). Similarly, scRNAseq analyses have provided new insights into the cancer‐type specific features of CAFs [21]. myCAFs have also been referred to as LRRC15+ CAFs or mCAFs [21, 60], and are generally considered fully differentiated collagen‐expressing myCAFs [21, 44, 49], by analogy to fully activated myofibroblasts [32]. Another relevant hallmark of LRRC15+ myCAFs is their strong immunosuppression [21, 22, 78]. Consistently, a subset of myCAFs expressing the cell surface serine protease FAP co‐localizes often with SPP1‐producing M2 macrophages [21, 22], which has been associated with T‐cell exclusion [21], suggesting a frequent pathologic myCAF‐macrophage crosstalk.

**Table 2:** Key transcriptional markers for CAFs subtypes.

	GENE	Ref cross‐tissue	Ref lung	Ref lung cancer metastasis
Myofibroblast‐like CAFs (myCAFs)	*LRRC15*	[21], [60]	[22], [44]	
	*CTHRC1*	[60]	[22], [44], [49]^a^	
	*MMP11*	[21], [60]	[79]	
	*POSTN*	[21], [32], [60]	[22], [49]^a^	
	*FAP*	[60]	[22]	[84]
	*COL1A1*	[32]	[49]^a^, [80]	[84]
	*COL1A2*	[32], [60]	[22]	
	*COL3A1*	[60]	[22], [49]^a^, [80]	[84]
	*COL5A1*	[60]		
	*COL10A1*	[21], [60]		
	*COL11A1*	[60]	[22]	
	*GREM1*		[22]	
	*BGN*		[22]	
	*TAGLN*		[22]	
Inflammatory CAFs (iCAFs)	*IL6*	[21], [60]	[79]	
	*CXCL1*	[21]		
	*CXCL12*	[21], [60]		[84]
	*PLA2G2A*	[60]		
	*CD34*	[60]		
	*CXCL14*	[60]		
	*PI16*	[60]		
	*APOD*	[60]		[84]
	*CFD*	[60]		
	*PRG4*	[60]		
	*C7*			[84]
	*CXCL2*			[84]
	*PDGFRA*			[84]
Antigen‐presenting CAFs (apCAFs)	*CD74*	[21], [60]		[84]
	*HLA‐DRA*	[21], [60]		[84]
	*HLA‐DRB1*	[21], [60]		[84]
	*CXCL12*	[21]		
	*RGS1*			[84]
	*CCL4*			[84]
proliferating CAFs	*STMN1*	[21], [60]		
	*RBP1*	[21]		
	*STAR*	[21]		
	*TUBA1B*	[60]		
	*MKI67*	[60]		
reticular CAFs	*CCL19*	[60]	[22]	
	*CCL21*	[60]	[22]	
	*VCAM1*		[22]	

^a^ In fibrotic conditions (not cancer).

There is also consensus that the most abundant subtypes (myCAFs and iCAFs) may be subclassified further, although an agreement on this subclassification is missing [19]. Part of this lack of consensus stems from the use of distinct classification criteria, with some groups favoring the spatial proximity to different tissue structures (vessels, tumor nests, and hypoxic/necrotic areas) [22, 60, 79], while others favor the *in silico* bioinformatic clustering of transcriptional data as indicative of different levels of activation, as in normal myofibroblasts [21, 32, 80]. On the other hand, the common abundance of myCAFs and iCAFs may simply indicate a dominant profibrotic or pro‐inflammatory microenvironment, respectively [17, 21]. Among them, the LRRC15+ myCAF phenotype elicited by profibrotic cues appears to be more homogeneous since it has been extensively reported in cancer [21, 44, 60] and fibrosis [49]. In contrast, the phenotypes elicited by inflammatory cues are more heterogeneous and context‐specific [21, 49, 60, 79], probably due to the variety and biological complexity of processes underlying pathologic inflammation, such as smoking, stress, injury, obesity, and hypoxia [7, 36, 55, 81, 82]. These diverse pro‐inflammatory contexts may be another major contributor to the lack of consensus on the subclassification of iCAFs as well as their disparate association with prognosis [36, 83, 84].

Regarding their origin, there is a consensus that most CAFs arise from the activation of local tissue‐resident fibroblasts [30], similarly to myofibroblasts in injured tissues and organ fibrosis [32]. Consistently, a cross‐tissue scRNAseq analysis supported that LRRC15+ myCAFs may arise from PI16+/COL15A1+ fibroblasts and pericytes [21]. Similarly, less activated myCAFs subtypes may arise from local fibroblasts exposed to a mixture of pro‐inflammatory and profibrotic cues [21]. Single‐cell analyses of NSCLC and other solid tumors have also reported CAFs exhibiting normal‐like traits in tumors and adjacent tissues, including molecular markers resembling those of PI16+ fibroblasts [21]. A higher abundance of these normal‐like CAFs in relation to LRRC15+ myCAFs has been associated with immunocompetent tumors [21, 22], supporting further that they may be major players in restraining tumor progression. However, it remains unknown whether tumor‐restraining and tumor‐promoting CAFs have different origins or rather reflect the context‐dependent evolution of the same pool of CAFs [18, 26], although current evidence supports the latter scenario [18, 21, 22]. In addition, other sources for CAFs have been described, including the recruitment of bone‐marrow‐derived fibrocytes and subsequent differentiation into fibroblast‐like cells [21, 30], the transdifferentiation of endothelial cells [21] and, to a lesser extent, the transdifferentiation of adipocytes and pericytes [30], although their relative contributions remain undefined.

### Controversies on key markers of myCAFs and iCAFs

While important agreements are emerging regarding major aspects of myCAFs and iCAFs, there are still relevant discrepancies and confounding issues that require further clarification. First, there is confusion regarding the expression patterns and functions of the most common markers used to identify those myCAFs exhibiting a strongly activated phenotype, including α‐SMA, FAP, and LRRC15. α‐SMA is a contractility marker that has been extensively used in myCAFs based on their functional analogy with the general myofibroblast phenotype reported in tissue repair and fibrosis [32, 33]. FAP has been favored by its more restricted expression patterns within the tumor stroma compared to α‐SMA [27], whereas LRRC15 has been more recently reported in scRNAseq analyses and associated with strong immunosuppression [21, 22, 78]. However, these three markers exhibit often distinct expression patterns and/or inconsistent functions. Thus, FAP+ CAFs can be either α‐SMA+ or α‐SMA‐, and vice versa [22, 60, 85]. Yet, there is consensus that α‐SMA+ CAFs are associated with bad prognosis in NSCLC [86]. Similarly, FAP+/α‐SMA+ CAFs are associated with poor outcome in NSCLC and with aggressive breast cancer subtypes [22, 76, 85]. In contrast, FAP‐ CAFs are generally associated with better prognosis, although there are some exceptions [86, 87, 88]. On the other hand, LRRC15 has a complex relation with collagen deposition, with data supporting that it may promote it or reduce it, depending on the context [45]. In addition, the levels of α‐SMA (protein) and the RNA of the gene that encodes it (*ACTA2*) exhibit often a weak correlation, suggesting that *ACTA2* may misclassify myCAFs in scRNAseq studies. Because fully activated myofibroblasts were initially defined based on their contractile and collagen‐producing functions [32], and for consistency with the fields of tissue repair and organ fibrosis, it is advisable to prioritize the expression of α‐SMA and fibrillar collagens to distinguish fully activated myCAFs from intermediate states or subtypes. Secondly, there is a disagreement regarding the interpretation of MYH11 protein or gene, with some authors using it as a marker of myCAFs [22, 60, 79, 80], whereas others assign it to smooth muscle cells [21]. Thirdly, there is a major overlap between the secretome of senescent fibroblasts and iCAFs, including IL‐6 production [30, 89], although the actual contribution of senescence in CAFs remains poorly explored.

## Specific molecular and phenotypic alterations of lung CAFs

**Fig. 2:**
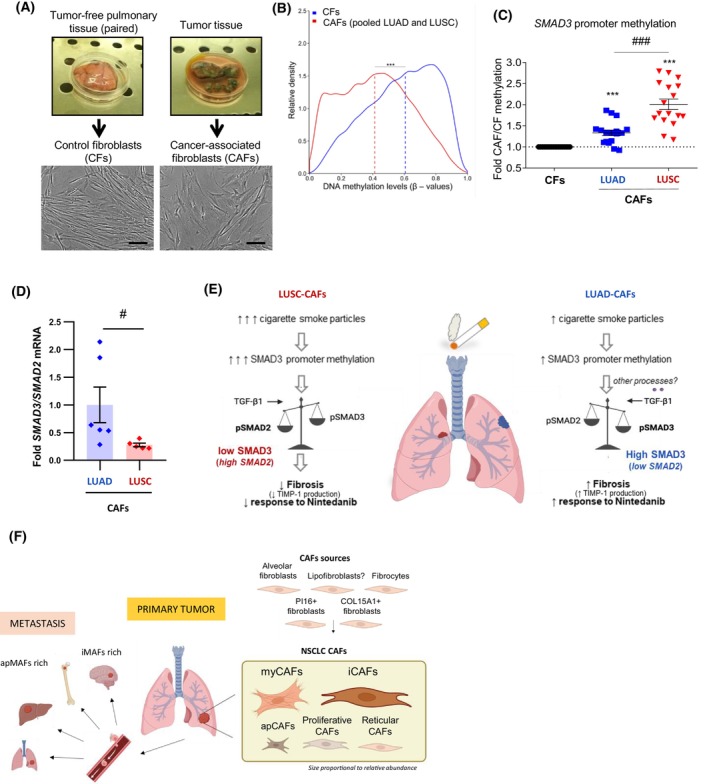
Relevant molecular alterations between lung CAFs and paired control fibroblasts (CFs) or between histotypes in LUAD and LUSC. (A) Representative images of tumor‐free pulmonary tissue (left) and a tumor tissue sample (right) collected from surgical lung cancer patients. Surgical pieces were used to isolate control fibroblasts (CFs) and paired CAFs, respectively. Reprinted from [24] with permission. Scale bar, 50 μm. (B) Histogram showing the relative density of average DNA methylation levels (*β*‐value) in CAFs (red line) and control fibroblasts (blue line). Vertical dashed lines show the median *β*‐values. Reprinted from [60] with permission. ***P < 0.001. (C) Fold (CAF/CF) SMAD3 promoter methylation of primary CAFs and their paired CFs (6 LUAD, 6 LUSC) determined by pyrosequencing in three CpG sites (used as technical replicates). Reprinted from [54] with permission. ***, *P* < 0.005 with respect to CFs; ###, *P* < 0.005; (D) Fold *SMAD3/SMAD2* mRNA ratio in LUAD and LUSC‐CAFs treated for 3 days with 2.5 ng·mL^−1^ TGF‐β1. Reprinted from [63] with permission. *#*, *P* < 0.05. (E) Emerging model on the histotype‐dependent relationship between exposure to smoking particles, SMAD3 promoter methylation, fibrosis, and response to antifibrotic drugs in lung CAFs. Reprinted from [54] with permission. (F) Summary of major sources and molecular subtypes of CAFs in primary tumors and metastatic settings in NSCLC.

Previous efforts to characterize CAFs in NSCLC in terms of epigenetic alterations, transcriptional profiling, protein expression patterns, metabolism, and ECM architecture have unveiled specific molecular traits and their dependencies on the histology. A genome‐wide DNA methylation profiling revealed that lung CAFs are epigenetically reprogrammed through global DNA hypomethylation compared to paired control fibroblasts from uninvolved pulmonary tissue (Fig. 2A,B) [62]. Moreover, lung CAFs exhibited epigenetic repression through DNA hypermethylation in selected genes, including *SMAD3* and *TBX4* [62]. The epigenetic repression of the pro‐fibrotic transcription factor SMAD3 was higher and functionally relevant in LUSC compared to LUAD (Fig. 2C), providing a straightforward mechanism for the lower fibrosis and poorer response to the antifibrotic drug Nintedanib of LUSC compared to LUAD reported in preclinical models and clinical trials [54, 90, 91]. Notably, exposing normal pulmonary fibroblasts to cigarette smoking particles condensed into culture medium was sufficient to elicit a time‐ and dose‐dependent increase in *SMAD3* DNA promoter methylation, whereas such epigenetic event did not occur in its closely related homolog *SMAD2*, thereby providing the first rationale for the puzzling epidemiologic observation that smoking is associated with a lower risk of radiotherapy‐induced pneumonitis/fibrosis in NSCLC [92]. These findings identified the ratio SMAD3/SMAD2 (at RNA or protein level) as a useful functional biomarker for myCAFs in NSCLC (Fig. 2D), since it is indicative of many histotype‐dependent alterations reported in lung CAFs [54, 65, 93] (Fig. 2E). On the other hand, TBX4 is an important regulatory transcription factor of the mesodermal progenitors that give rise to nearly all mesenchymal cells in the adult lung, which supply the myofibroblast population upon injury and pulmonary fibrosis [94, 95]. Intriguingly, TBX4 was found downregulated in lung CAFs [96] through epigenetic repression [62], which may compromise their progenitor potential, although the pathologic impact of such *TBX4* repression remains to be determined. In addition to DNA methylation changes, the reprogramming of lung fibroblasts into CAFs involves alteratered expression patterns in non‐coding RNAs, including the downregulation of miR‐16 to enhance HGF secretion [97]. Additional epigenetic alterations are reviewed elsewhere [24].

**Fig. 3:**
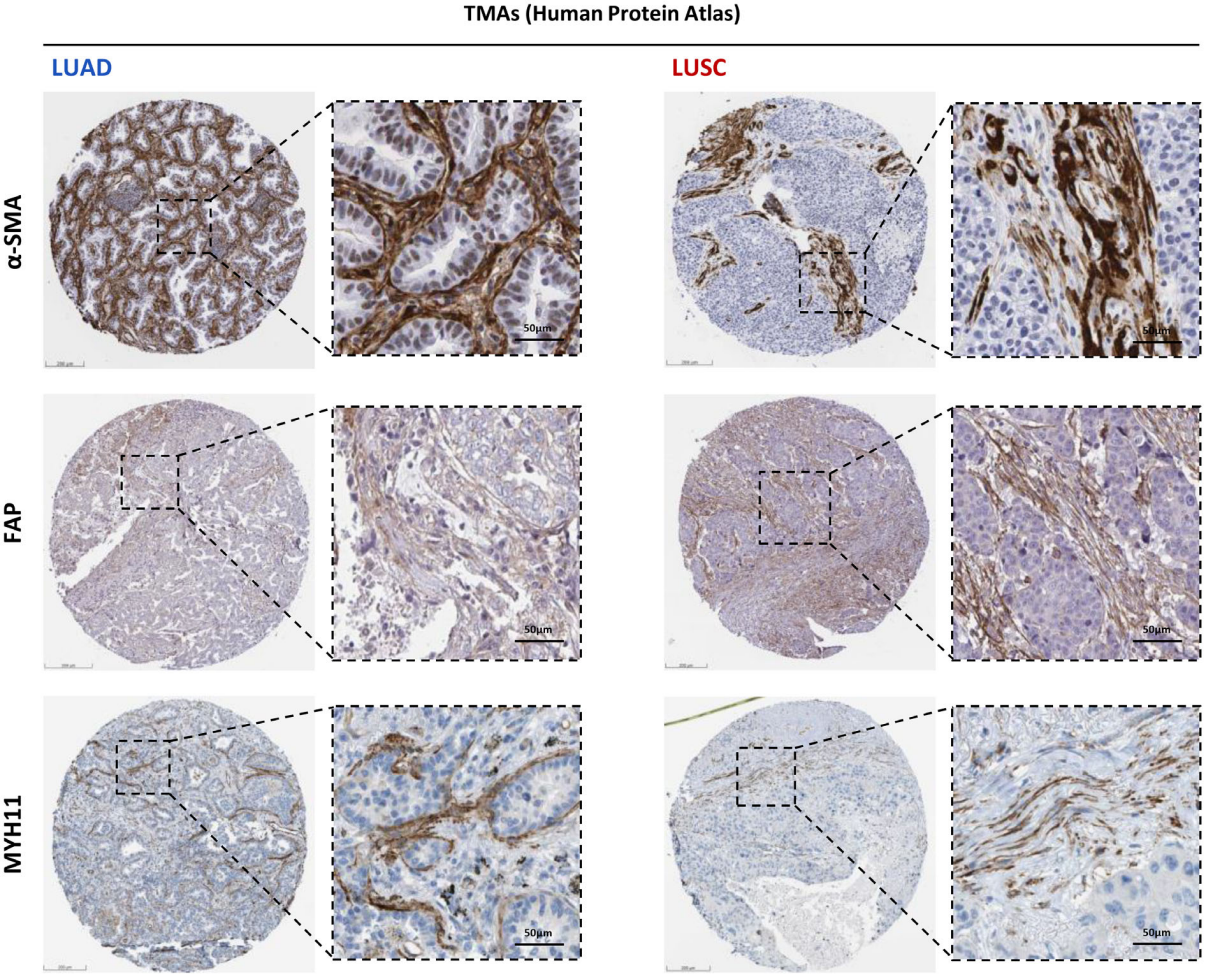
Differential expression of major myCAF markers in LUAD and LUSC. Representative whole tissue and zoom images of α‐SMA (top), FAP (middle), and MYH11 (bottom panels) histological stains within tissue microarrays of LUAD and LUSC downloaded from the Human Protein Atlas (HPA) database. Scale bar, 200 μm.

Regarding their major subtypes, LRRC15+ myCAFs are dominant in NSCLC [22, 44, 79] (Fig. 2F); yet there is evidence that α‐SMA+ myCAFs abundance and expression of fibrillar collagens and other fibrosis markers are higher in LUAD than LUSC, particularly in smokers [54, 79]. Consistently, higher glucose uptake was found in CAFs from LUAD patients compared to LUSC [98], which could account for the metabolic demands required for the increased collagen deposition reported in LUAD. Similarly to α‐SMA+ myCAFs, FAP+ myCAFs are rather ubiquitous in NSCLC, exhibiting variable expression of α‐SMA, although they are most abundant in late stages, LUSC, and solid type LUAD [22] (Fig. 3). In addition to increased collagen deposition, LUAD‐CAFs upregulate the production of tissue inhibitor of metalloproteinase‐1 (TIMP‐1), which elicits important tumor‐promoting cytokine‐like functions [93] and promotes drug resistance to Nintedanib [90] (Fig. 2E). The outcome of dysregulated ECM homeostasis in NSCLC is a shift towards increased deposition and marked remodeling of fibrillar collagens through longer, straighter, and more aligned fibers, which is more evident in LUAD [99] (Fig. 1A,B). Notably, lung myCAFs have been consistently associated with poor prognosis and immunosuppression, particularly FAP+/α‐SMA+ CAFs [22, 79, 85]. Likewise, α‐SMA was significantly associated with increased risk of death or disease progression in a meta‐analysis, whereas FAP was not [86]. In contrast, FAP‐ CAFs were associated with better prognosis regardless of α‐SMA status in a large lung cancer patient cohort [85]. In this context, it would be desirable to clarify the histotype relationship between the SMAD3/SMAD2 ratio, α‐SMA, and FAP.

A subtype of CAFs characterized by high MMP1 expression exhibiting characteristics of both myCAFs and iCAFs was reported in a cross‐tissue scRNAseq analysis in different cancer types, including lung cancer. These MMP1+ CAFs exhibited strong immunosuppression and resistance to immunotherapies [21]. Intriguingly, high MMP1 expression has been reported in a rare and aggressive NSCLC subtype called large cell carcinoma, which is rich in both poorly differentiated cancer cells and senescent CAFs that upregulate many pro‐inflammatory factors [89]. Of note, co‐stimulating normal pulmonary fibroblasts with MMP1 and TGF‐β1 was sufficient to elicit a senescent‐like phenotype, revealing a novel pathologic function of excessive MMP1 production in cancer. However, the actual abundance of MMP1+ CAFs in LUAD and LUSC remains undefined. On the other hand, as in α‐SMA+ myCAFs, iCAFs were reported to be more abundant in LUAD than LUSC, yet they have been largely associated with longer overall survival and weaker dissemination.

A population of fibroblasts positive for ADH1B and some markers of normal/progenitor fibroblasts (PI16+) and alveolar fibroblasts has been described in early‐stage tumors, and has been associated with immunocompetence and better prognosis, particularly when they outnumbered LRRC15+ myCAFs [21, 22]. Moreover, the origin of most myCAFs appears to be PI16+ fibroblasts and alveolar fibroblasts [21, 22, 44], although their contributions to myofibroblasts in pulmonary fibrosis remain controversial [44, 47]. Likewise, PI16+ fibroblasts are suspected to be major contributors to iCAFs [79]. In contrast, MMP1+ CAFs may arise from COL15A1+ fibroblasts [21], although direct evidence of COL15A1+ fibroblasts contributions to lung CAFs is scarce. On the other hand, the contribution of lipofibroblasts to lung CAFs is unknown, although adipocyte‐like mesenchymal cells resembling lipofibroblasts may contribute to CAFs in other cancer types [30]. Conversely, there is direct evidence that fibrocytes contribute to the overall population of lung CAFs [62], although their relative presence is unknown (Fig. 2F). Fibrocytes are bone‐marrow‐derived monocytic cells, and recent work indicates that they can contribute to the tumor‐supportive niche in lung cancer through the endothelin system [100].

In addition to the universal α‐SMA+ or FAP+ myCAFs (Fig. 3, top‐middle panels) and iCAFs subtypes, an mIHC study revealed an enrichment of a subpopulation of MYH11+/α‐SMA+ CAFs in early‐stage NSCLC tumors, whose origin remains unknown. Notably, this CAF population was often found lining tumor nests selectively in LUAD of acinar/papillary histologic subtype, but not in solid subtype LUAD (associated with poor differentiation) or LUSC (Fig. 3, bottom panels). Moreover, MYH11+/α‐SMA+ CAFs correlated with T‐cell exclusion and high collagen deposition as in FAP+/α‐SMA+ CAFs, although both subtypes differed in their molecular patterns of collagen production [22]. The latter mIHC study further reported that NSCLC tumors were generally dominated by either ADH1B+ or FAP+ CAFs, and that FAP+ CAFs were particularly associated with SPP1‐producing macrophages in LUAD [22], although independent analysis in larger cohorts should confirm these observations. In addition, single‐cell imaging mass cytometry of a large patient cohort revealed an enrichment of other CAF populations in LUSC, including podoplanin (PDPN+) CAFs and hypoxic CAFs (CAIX+), which were associated with poor prognosis or high risk of relapse [79]. Consistently, PDPN+ CAFs were strongly associated with poor prognosis in a meta‐analysis [86], and are emerging as important regulators of tumor immunity [101]. A summary of the common markers of major emerging lung CAF subtypes is shown in Table 2. Collectively, these recent single‐cell analyses refine the initial observation of histotype‐dependent phenotypic alterations in lung CAFs reported a decade ago [16], and begin to delineate how CAF heterogeneity in NSCLC depends on the stage and histologic subtype.

## Metastatic stromagenesis and metastasis‐associated fibroblasts (MAFs): focus on the brain

### General features of stromagenesis and fibroblasts in metastasis

Metastasis accounts for most cancer‐related deaths [102]. Pathologists have long recognized that metastasis resembles key aspects of the primary tumor, including a desmoplastic stroma rich in activated myofibroblasts [103], supporting that successful metastases also require a tumor‐promoting or tumor‐permissive stroma [104]. This resemblance probably contributed to the success of AI‐based digital pathology tools to identify the origins of metastatic tumors of unknown primary [105]. Fibroblasts in these metastatic sites are referred to as metastasis‐associated fibroblasts (MAFs) and remain much less characterized than primary CAFs. Likewise, both the mechanisms underlying the formation of the activated stroma in metastatic sites and the sources of MAFs are poorly defined, yet analogous processes to primary tumors are commonly assumed, including local tissue‐resident fibroblasts as the main source of MAFs [23]. Alternatively, CAFs may co‐travel with disseminating cancer cells as part of circulating tumor cell (CTCs) clusters [23, 106, 107] (Fig. 2F), which are more effective in setting metastatic colonies than single CTCs [103, 106]. In breast cancer, CD44 is an important mediator of the heterotypic clustering of CAFs and CTCs [106], but the overall mechanisms of these heterotypic interactions and their relevance in other solid tumors remain largely unknown. In support of this co‐traveling process, CAFs were reported in CTC clusters in the blood of breast cancer patients [104, 107] and in *in vivo* models of breast cancer [108, 109], concomitantly with enhanced metastases [104, 106, 110]. However, additional experimental and clinical validation is required. Beyond supporting colonization at metastatic sites, CAFs may actively contribute to the increase in the population of metastasis‐initiating cells by promoting EMT and/or stem‐like traits in cancer cells [17, 28].

The current definition of MAFs is equivalent to CAFs in metastatic settings [23]. In further analogy to CAFs, MAFs are heterogeneous [111], and scRNAseq analyses have identified markers reminiscent of myCAFs, iCAFs, and apCAFs [84], which can be referred to as myMAFs, iMAFs, and apMAFs, respectively, for consistency. As in primary tumors, myMAFs are the most abundant subtype, and either α‐SMA+, FAP+, or α‐SMA+/FAP+ myMAFs have been reported in brain metastasis from numerous cancer types, including lung carcinomas [104, 111]. Similar observations have been reported in *in vivo* models [104]. Regarding their function, MAFs promote the establishment of metastatic niches and mediate therapy resistance through overlapping processes with CAFs, although a MAF‐specific overproduction of factors that promote angiogenesis and/or EMT has been suggested [23], which may be particularly crucial in facilitating metastatic spreading. Even though the mechanisms of the pathologic effects of MAFs are ill defined, some studies have also highlighted the role of SPP1 [84, 110].

### Specific features of stromagenesis and MAFs in NSCLC

NSCLC is the most common source of brain metastases [112], although it can also disseminate to the bone, lung, and liver [5, 6]. Notably, scRNAseq analysis of bone, brain, and intrapulmonary metastasis from lung cancer patients identified clusters of myMAFs, iMAFs, and apMAFs; yet they found a larger presence of iMAFs in the brain, whereas apMAFs were dominant in bone metastasis [84] (Fig. 2F). This study also reported a common association with immunosuppression through Treg crosstalk, in which SPP1 appeared to play an important role. Another scRNAseq study comparing CAFs and brain MAFs in LUAD reported an enrichment in myofibroblast‐like cells with higher angiogenic capacity concomitantly with lower infiltration of immune cells in metastatic settings [113, 114].

MAFs in lung cancer brain metastasis can express α‐SMA, FAP, and, to a lesser extent, both markers [104, 111]. Intriguingly, although FAP expression has been largely reported in MAFs within collagen‐containing perivascular and septal regions, it was also found in a fraction of metastatic cancer cells [111, 115], which may complicate the identification of MAFs in scRNAseq analysis based on FAP. There is also evidence that reactive astrocytes may contribute to the altered stroma within brain metastasis (21), by analogy to their active role in physiologic scar formation within the brain [116]. However, the relationship between activated astrocytes and MAFs remains ill defined. Some authors have examined also the contributions of pericytes in brain metastasis, which are involved in scar formation upon brain injury [117]. Indeed, both astrocytes and pericytes are integral components of the brain–blood barrier [118], and are therefore among the first cell types that CTCs interact with during the formation of early brain metastasis. However, there are conflicting observations on the pathologic potential of pericytes in lung cancer brain metastasis, including protective [119] or stimulating [120] roles. Collectively, our understanding of stromagenesis and MAF heterogeneity in the metastatic dissemination of NSCLC is still very limited, owing in part to the lack of suitable preclinical models and reduced availability of patient samples.

## Open questions and challenges

Our knowledge of stromagenesis and CAF heterogeneity in NSCLC and other solid tumors has accelerated in recent years, yet there are major remaining unanswered questions and technical challenges that future research should address. Regarding stromagenesis, it remains poorly understood how tumors build their large accumulation of CAFs and how the tumor stroma switches from tumor‐restraining to tumor‐promoting. Likewise, how pathologic inflammation/stress, epigenetic reprogramming, and dysfunctional tissue repair converge to drive chronic CAF activation is ill‐defined. The lack of suitable preclinical models and clear markers to address these questions is a major limitation of the field [26]. Regarding CAFs, their current classification is largely based on transcriptional data gathered from scRNAseq studies. However, this technique has important limitations, including the aggressive methods used to detach fibroblasts [30] and the requirement for protein and functional validations [26]. These validations are particularly needed for myofibroblast‐like CAFs, since myofibroblasts were originally defined based on their enhanced contractility and collagen‐producing functions, and this functional definition remains the standard in related fields (i.e. tissue repair and organ fibrosis) [32]. However, contractility cannot be directly inferred from transcriptional signatures in scRNAseq. Moreover, it remains unclear whether the current classification of CAFs represents either bona fide subtypes, their dynamic adaptation to the local microenvironment, or different maturation states. Future work should clarify how many truly distinct CAF subtypes exist, which are conserved across cancer types or are rather cancer type/subtype‐specific (as in LUAD versus LUSC), what their key functional roles are particularly in terms of immune regulation and other cancer‐relevant processes, and what their origins are.

In metastatic settings, more research is clearly needed to clarify the sources and tumor‐promoting functions of MAFs. Future consensus regarding biomarkers and notation for CAFs and MAFs is also required to add clarity to this field, although α‐SMA stands out as an important protein biomarker for myCAFs/myMAFs, given its traditional use to identify contractile myofibroblasts and its strong immunosuppressive features. In NSCLC, future work should expand our understanding of stromagenesis and CAFs/MAFs heterogeneity, including their histologic dependencies.

There is growing interest in exploiting CAF‐related alterations to overcome therapy resistance and develop new therapeutic approaches in NSCLC [27, 57, 90, 121] and other cancer types [25, 26]. Advances in this arena are expected to help define new therapeutic strategies to target or reprogram tumor‐promoting CAFs and MAFs, or to expand their tumor‐restraining counterparts. These fibroblast‐oriented therapeutic approaches hold great promise, particularly in combination with chemotherapy and immunotherapy.

## Conflict of interest

The authors declare no conflict of interest.

## Author contributions

AB and JA conceptualized the work, wrote the main part of the original draft, and prepared the figures and tables. ND‐V wrote a section of the manuscript. PF‐N revised and edited the manuscript. All authors have read and agreed to the published version of the manuscript.
